# Supplementary material for: Complexome profiling of the Chlamydomonas psb28 mutant reveals TEF5 as an early PSII assembly factor
Source: Plant Cell. 2025 Mar 17;37(6):koaf055. doi: 10.1093/plcell/koaf055 (PMC12164590; doi:10.1093/plcell/koaf055)
Supplement: koaf055_Supplementary_Data [file koaf055_supplementary_data.zip › psb28_interactive.zip!/plots/intensity/qualityPlots/peptides/QCR8_Cre03.g156950.t1.1.html]

### Cre03.g156950.t1.1

Trivial name: QCR8  
SeqCoverage [median]: 54.80  
Mass: 8.67 kDa  
  
 

| Sequence | MissedCleavage | Mass [Da] | wtIntensity | muIntensity | Score |
| --- | --- | --- | --- | --- | --- |
| EILYQLSPYQQDVIR | 0 | 1863.97 | 19213.85 | 0.00 | 217.83 |
| GYTEHEMHQER | 0 | 1415.59 | 14993.30 | 14213.06 | 126.71 |
| QNIPLR | 0 | 739.43 | 7764.69 | 1917.30 | 106.84 |
| QNIPLREILYQLSPYQQDVIR | 1 | 2585.40 | 1538.59 | 0.00 | 74.03 |
| QTFTNAPK | 0 | 905.46 | 28349.50 | 22147.75 | 142.36 |
|  |  |  | 71859.9 | 38278.1 |  |
